# Explainable Depression Detection Based on Facial Expression Using LSTM on Attentional Intermediate Feature Fusion with Label Smoothing

**DOI:** 10.3390/s23239402

**Published:** 2023-11-25

**Authors:** Yanisa Mahayossanunt, Natawut Nupairoj, Solaphat Hemrungrojn, Peerapon Vateekul

**Affiliations:** 1 Department of Computer Engineering, Faculty of Engineering, Chulalongkorn University, Phayathai Rd, Pathumwan, Bangkok 10330, Thailand; 6470165021@student.chula.ac.th (Y.M.); natawut.n@chula.ac.th (N.N.); 2 Center of Excellence in Digital and AI Innovation for Mental Health (AIMET), Chulalongkorn Unversity, Phayathai Rd, Pathumwan, Bangkok 10330, Thailand; 3 Department of Psychiatry, Faculty of Medicine, Chulalongkorn University, Phayathai Rd, Pathumwan, Bangkok 10330, Thailand; 4 Cognitive Fitness and Biopsychiatry Technology Research Unit, Faculty of Medicine, Chulalongkorn University, Phayathai Rd, Pathumwan, Bangkok 10330, Thailand

**Keywords:** depression detection, facial expression, deep learning

## Abstract

Machine learning is used for a fast pre-diagnosis approach to prevent the effects of Major Depressive Disorder (MDD). The objective of this research is to detect depression using a set of important facial features extracted from interview video, e.g., radians, gaze at angles, action unit intensity, etc. The model is based on LSTM with an attention mechanism. It aims to combine those features using the intermediate fusion approach. The label smoothing was presented to further improve the model’s performance. Unlike other black-box models, the integrated gradient was presented as the model explanation to show important features of each patient. The experiment was conducted on 474 video samples collected at Chulalongkorn University. The data set was divided into 134 depressed and 340 non-depressed categories. The results showed that our model is the winner, with a 88.89% F1-score, 87.03% recall, 91.67% accuracy, and 91.40% precision. Moreover, the model can capture important features of depression, including head turning, no specific gaze, slow eye movement, no smiles, frowning, grumbling, and scowling, which express a lack of concentration, social disinterest, and negative feelings that are consistent with the assumptions in the depressive theories.

## 1. Introduction

Today, Major Depressive Disorder (MDD) is a widespread condition that affects people all over the world. Suicide and life-threatening situations can result from this illness. Furthermore, the effects of COVID-19 make this problem worse [1,2,3,4,5]. During the epidemic, the number of depressed patients is rising rapidly. A fast diagnosis and receiving the appropriate care are essential to reducing the risk of life-threatening depression. However, the majority of people are unaware of their disease and unable to access appropriate medical care. On the other hand, all citizens have insufficient access to medical staff and therapy. Artificial intelligence can therefore be used to support medical care as a primary decision tool or as a decision-support tool.

Clinical interviews [6] are one technique for diagnosing depression. The diagnosis procedure includes a list of questions that might judge anhedonia, which is the inability to experience pleasure; anergia, which is a persistent feeling of being run down; focus; appetite; sleep; guilt; and suicide. During interviews, the patient’s expression, posture, voice tone, and content of their answers are scrutinized. Using facial expression and body language, a psychiatrist can identify depression. Similar to a psychiatrist’s observation, artificial intelligence is capable of recognizing facial expressions and body language without the usual ambiguity or language barrier. As a result, the focus of this study is solely on face expression.

**Figure 1:**
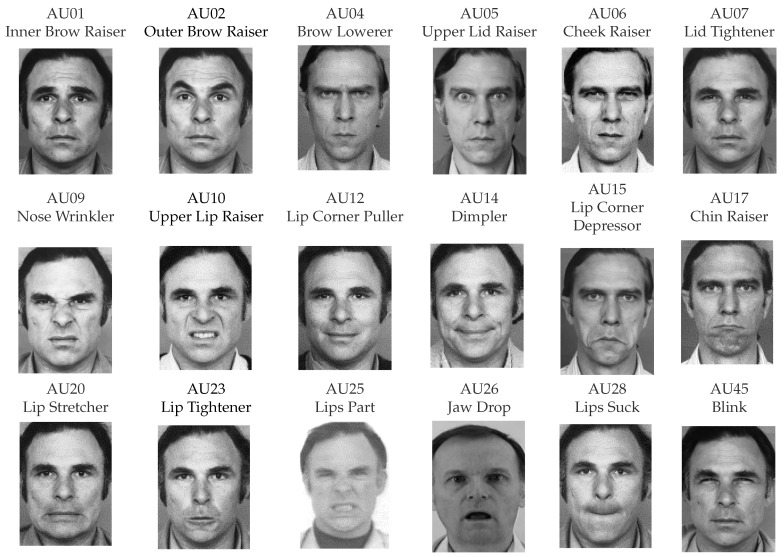
Example of action units.

The Facial Action Coding System (FACS) [7], which describes the relationship between muscle use and emotion in a coding system, defines facial expression. The example of an action unit in a coding system is shown in Figure 1. This approach defines not only facial emotion but also head posture and eye contact, which reflect entire facial movements. This system’s ability to convert video input to numerical data makes it incredibly helpful for machine learning models.

Openface [8] is a piece of artificial intelligence that may be used to recognize facial expressions that are encoded in the action units coding system. The open-source Openface tool can estimate head posture, identify facial action units, and estimate eye gazing. This tool is used to extract data on head posture, facial action units, and eye gaze for use as input in machine learning models. Compared to video data, the extracted data is more lightweight. As a result, the machine learning model is compact and portable. In order to quickly identify non-psychiatrist depression, we plan to use a machine learning model that mimics methods for diagnosing depression through the observation of facial expressions.

To resolve this issue, we employed the tools listed above to assist medical professionals in developing machine learning models that accurately identify depression without interference from privacy concerns or language barriers. Additionally, we aimed to clarify the major components that it discovered by using an explainable approach to the model in order to make the model’s essential features more comprehensible to humans and to increase our understanding of the association between depression and facial expression. Our main contributions are listed as follows:

- In order to choose the model that will perform the best, we evaluated three types of fusion modeling techniques that use the transformer model, window block LSTM, and Bi-LSTM. In addition, we used feature selection techniques and label smoothing techniques to improve model accuracy.
- Data used in this study are extracted from clinical interview videos using Openface tools in time-series format. Data are classified as depressed or non-depressed.
- We apply an integrated gradient [9] over the model and sort the significant features in order to describe the main characteristics of facial expression that can detect depression.

The rest of the paper is structured as follows: The related works are summarized in Section 2. How to prepare the data set and the proposed methods used in this study are explained in Section 3. Section 4 presents the results and discussion of the proposed methods. Finally, the summary of the study is provided in Section 5.

## 2. Related Work

The brain and nervous system link depression and facial expression. The relationship between facial expression and the brain and nervous system has been studied using electroencephalographic (EEG) studies [10]. The findings demonstrate that EEG analysis can identify the pattern of muscle use when a person displays an emotion on their face, such as a grin, rage, or sadness. the same way as an experiment that makes use of fMRI (functional magnetic resonance imaging). By using fMRI analysis, the two investigations [11,12] investigate how depressed patients and non-depressed patients respond to happy and sad faces, respectively. The results of the fMRI investigation show that depressed patients’ brains react differently from healthy individuals to sad faces compared to happy faces. Despite the fact that people rarely exhibit their emotions in regular situations, during clinical interviews, people frequently utilize their faces to convey their true feelings because speaking and facial expression go together in one [13]. On the other hand, people can tell the difference between depressed patients and regular people just by looking at them [14]. In a similar vein, artificial intelligence can spot depression while performing cognitive tasks [15]. Reduced mouth or eye movements while performing the task are the observed points. There is evidence from earlier studies that people or machine learning models may identify depression by studying a particular facial expression.

The use of artificial intelligence in diagnosing psychiatric diseases is currently expanding. The input information and methods used to diagnose psychological problems can be clearly divided into three and five categories, respectively [16]. MRI, EGG, and kinesics diagnosis (which includes behavioral, facial, and other physical data) are the three primary inputs. Bayesian models, logistic regression, decision trees, support vector machines, and deep learning are five strategies. In this study, we used deep learning approaches (LSTM and transformer model) to improve the performance of kinesics diagnosis, which includes facial expression, head pose, and eye gaze to diagnose depression.

As shown in Table 1, there are numerous methods for identifying depression or categorizing its severity in recent years. The majority of them make use of every human behavior modality, such as video, voice, and speech content text, to input and pass through multiple models to improve performance. Detecting Depression with AI Sub-Challenge (DDS) of the Audio/Visual Emotion Challenge and Workshop (AVEC 2019) [17] is a well-known challenge that explores depression identification. This sub-challenge included an E-DAIC data set with extracted facial feature data and voice and speech content text. This sub-challenge’s winner is a multi-level attention network utilizing text, audio, and video for depression prediction [18]. They succeed in obtaining a concordance correlation coefficient (CCC) of 0.67 using a multi-model of three modalities. With a CCC of 0.733, the multi-transformers model is also applied to the E-DAIC data set [19]. The input of the multi-transformers model is the voice and facial features. The proposed approach combines the PHQ-8 regression label method and the PHQ-8 classification at five levels for multi-task learning. The DAIC-WOZ data set was used as input in the proposed algorithms that only concentrate on facial features. By employing particle swarm optimization (PSO) [20] to choose the best predictors of AUs, one proposed strategy focuses on minimizing AUs in a feed-forward neural network (FFNN). The most accurate predictors are AU04, AU06, AU09, AU10, AU15, AU25, AU26, AU04, AU12, AU23, AU28, and AU45. They have an accuracy rate of 97.83 percent. Another set of proposed methods reduced and selected facial features from the DAIC WOZ data set using Fisher Discriminant Ratio (FDR) and Incremental Linear Discriminant Analysis (ILDA) [21]. The best performance in the DAIC WOZ data set for their technique is an F1 score of 0.805. Based on facial features, movement intensity, speech, and text with 81 patients’ personal collection data sets, studies about posttraumatic stress disorder (PTSD) and major depressive disorder (MDD) have been conducted [22]. According to the results, the classification of MDD was 0.86 accurate, while the classification of PTSD was 0.9 accurate.

The limitations of all modality studies are privacy concerns and language barriers. The most frustrating aspect of all modality studies is the language barrier. Even so, the model may perform well as a result. Because of the numerous languages, it cannot be used by many people worldwide. In addition, the researchers found it difficult to acquire the large data set and were unable to share it among each other or obtain it from a public source because of privacy concerns. Due to these restrictions, machine learning models were difficult to use and enhance. Therefore, we may overcome challenges such as a language barrier and privacy concerns by focusing on facial expression modalities in Openface version 2.

## 3. Materials and Methods

**Figure 2:**
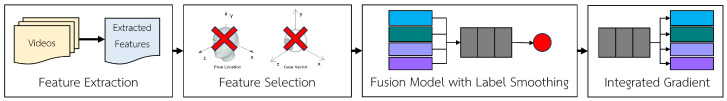
Overall proposed methods.

The proposed methods are shown overall in Figure 2. In feature extraction, we first extract videos from time-series extracted files, and then in feature selection, we choose some of the features. To evaluate performance, selected features are run through multiple fusion models with label smoothing. The best performance model is applied to an integrated gradient for the visualization of key features.

### 3.1. Input and Target Preparation

We used input normalization to enhance a network’s convergence properties. The longest input time series is not more than 11 min. Since there are 11 min, 60 s, and 30 frames per second, the timestep is 19,800. To fit this timestep, zero was added to input that falls under 11 min.

**Figure 3:**
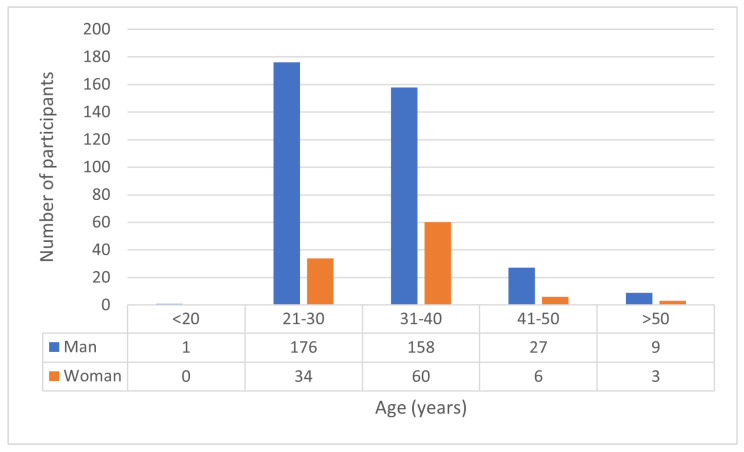
Gender and age diversity of the data set.

Figure 3 shows the diversity that was estimated by Deepface [23]. Because of the privacy protection policy of the data collection protocol, the private information of participants is not collected. Therefore, we decided to utilize Deepface to estimate the data set. The raw data includes levels of depression that are normal, mild, moderate, and severe. The levels are classified by the Thai version of the 7-item Hamilton Depression Rating Scale (HAMD-7) [24]. The Thai version of HAMD-7 is translated and reviewed by five psychiatrists. The normal level means no depressive symptoms. People can do their daily activities as normal. The mild level means depressive symptoms can be noticeable and interfere with daily activity. For instance, insomnia, weight loss, and irritability. The moderate level means that depressive symptoms cause problems with self-esteem, productivity, sensitivities, excessive worrying, and feelings of worthlessness. The symptoms of severe depressive levels are hallucinations, suicidal thoughts, or behaviors. We choose to distinguish between the depressed and non-depressed by not classifying them as having depressive levels because facial features have a slight depression cue. In order to apply the model as a binary categorization, we define normal and mild as non-depressed and moderate and severe as depressed.

The raw data has 106 normal, 234 mild, 112 moderate, and 22 severe. It is divided into three independent data sets for training, development, and testing in the ratio 80:10:10. The mild and normal categories are categorized as non-depression. The moderate and severe categories are categorized as depression. Data balancing is achieved by duplicate non-depressed classes in the train data set, as shown in Table 2.

### 3.2. Features Extraction

**Figure 4:**
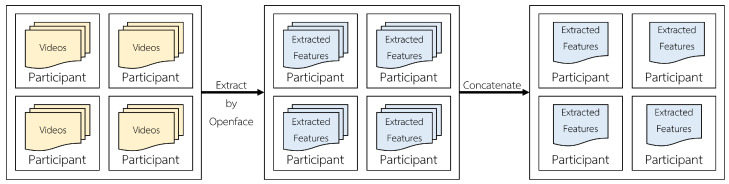
Our feature extraction.

The feature extraction method from videos to the extracted features file used as the model’s input is shown in Figure 4. Participants must respond to numerous questions in a clinical interview. However, it only records one participant’s response per video. Consequently, there are multiple videos for each participant. The OpenFace tool extracts participant videos. Following data extraction, we must join time-series extracted feature files from the same participant so that they can be used as model input. A time-series extracted features containing head posture, gaze, and action units was used.

### 3.3. Data Preprocessing

#### 3.3.1. Features Selection

Extracted features include the head posture with three rotational features (pitch, roll, and yaw) and three location features (x, y, and z axes), the gaze with six vector features (x, y, and z axes for both eyes) and two radian features (angle x, y), and the action units with 18 presence feature groups and 17 intensity features. The total number of features in the extracted features is 49. However, similar features can be removed in order to eliminate redundant data and model size.

**Figure 5:**
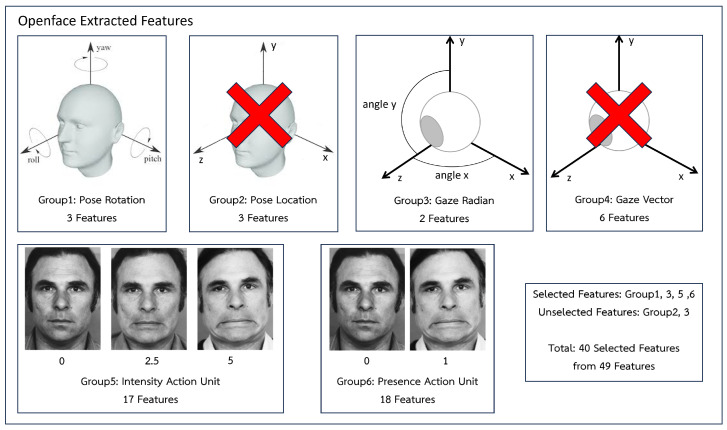
Our feature selection.

Similar to how the gaze feature has vector and radian features, the head posture feature has rotation and location feature groups that are repeated with different units, as shown in Figure 5. To compare their results, we experimented with them using a single model. The findings, as shown in Table 3, indicated that the head posture rotation group and the gaze radian feature group have good performance in classifying depression. The input for the model is therefore chosen from head posture rotation and gaze radian features. The action units feature has presence feature groups and intensity feature groups, all of which use distinct estimation models. Both feature groups are consequently chosen. In summary, three location features of head posture and six vector features of gaze are eliminated. Out of 49 features, the selected features contain 40 features.

#### 3.3.2. Label Smoothing

Label smoothing [25] is a regularization technique that can improve the performance of machine learning models, particularly in the training of neural networks for classification tasks. Label smoothing can prevent a model from becoming overconfident in its predictions because the true distribution of the data is not so clear-cut. Label smoothing addresses this by introducing a small amount of uncertainty into the labels. This method has the efficiency to improve our model since depressive syndrome is not clearly determinable, especially at a mild and moderate level. The formula for label smoothing is shown in Equation (1). $y_{k}^{LS}$ is a soft label. $y_{k}$ is a hard label. α is a label-smoothing number that should be in the range of 0 to 1 for smooth labels in range 1 to 0.5. *K* is the number of classes. The hard label in classification tasks, for instance, is always binary in nature and is [0, 1]. 0 denotes false, whereas 1 denotes true. Label smoothing causes soft labels to have probabilities between [0.5, 0.95] after applying. To reduce computation time, we chose to employ label smoothing with α range of 0 to 0.9 with 0.1 increments solely on the best model.

(1) $$y_{k}^{LS}=y_{k}\left(1-\alpha \right)+\alpha /K$$

### 3.4. Model Architecture

We chose to use fusion techniques to merge specific facial features in order to enhance the performance of the facial feature model. There are various fusion technique combinations, however. The early fusion technique, the intermediate fusion technique, and the late fusion technique are the three basic fusion techniques that will be used in this study. The Window Box LSTM model, Transformer model, and Bi-LSTM model are the three models that are assigned to each technique for assessing performance.

#### 3.4.1. Early Fusion Model Architecture

**Figure 6:**
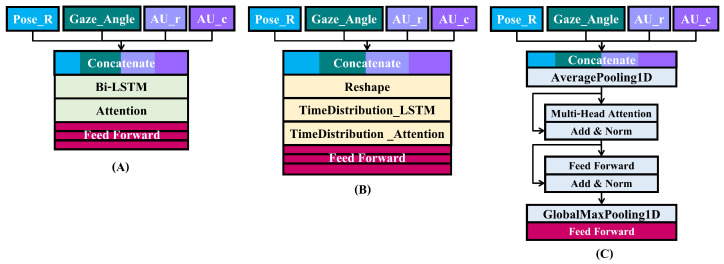
Early fusion model architecture. (**A**) Early Bi-LSTM fusion model architecture. (**B**) Early window block LSTM fusion model architecture. (**C**) Early transformer fusion model architecture.

Early fusion models combined features using a concatenate layer, which was able to transmit all 40 features to a single model. The following layers are shown in Figure 6A–C, respectively, for Bi-LSTM, Window Block LSTM, and Transformer. Following the concatenate layer, the Bi-LSTM model has 128 hidden units: the Bi-LSTM layer, attention layer, and feed forward layers, which have 64, 32, and 1 units. Because data are collected at a rate of 30 frames per second, the Window Block LSTM model must pass through reshape layers that reshape to (sample number, 660, 30, 40). The following layers include 64 hidden units of LSTM with time distribution, attention with time distribution, and feed-forward layers, which have 32, 16, and 1 units. Transformer models utilize 1 transformer block, 512 head sizes, 1 head number, and 2048 feed-forward dimensions. To shorten the time-series range before passing through the transformer model, an average pooling layer was employed for average sampling every 15 frames because the time-series are too long for the model.

#### 3.4.2. Intermediate Fusion Model Architecture

**Figure 7:**
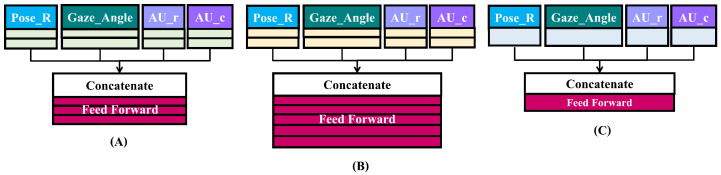
Intermediate fusion model architecture. (**A**) Intermediate Bi-LSTM fusion model architecture. (**B**) Intermediate window block LSTM fusion model architecture. (**C**) Intermediate transformer fusion model architecture. Green blocks refer to Figure 6A Bi-LSTM and attention layers. Yellow blocks refer to Figure 6B reshape, time distribution LSTM, and time distribution attention layers. Gray blocks refer to Figure 6C average pooling 1d and transformer layers.

Before concatenating layers and feeding forward layers, the intermediate fusion model uses its own layer and attention layer for each feature. Figure 7A–C display three different types of models. Bi-LSTM layers Pose, Gaze, AU_r, and AU_c have 64, 16, 128, and 128 hidden units, respectively, in the Bi-LSTM model. There are 128, 64, 1 units in the fefeed-forwardayers that follow. The LSTM layers of Pose, Gaze, AU_r, and AU_c for Window Block LSTM have 32, 32, 64, and 64 hidden units, respectively. The following feed-forward layers contain 128, 64, 32, 1616, and units. For each feature, the transformer model is the same as the early fusion model. The difference is that a concatenated layer is applied before the feed-forward layer.

#### 3.4.3. Late Fusion Model Architecture

**Figure 8:**
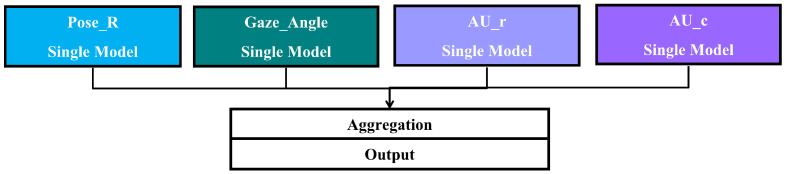
Late fusion model architecture.

The late fusion model uses a single model for all features and uses an aggregate layer to average the prediction values across every attribute before reaching a conclusion. The diagram of the late fusion model is displayed in Figure 8. Before feed-forward layers, the configuration of the model is the same as in the intermediate fusion model. The difference is the power of two in units of feed-forward layers.

### 3.5. Integrated Gradient Explanation

Integrated Gradient (IG) [9] is an explainability technique used to understand the relative of input features and predictions of machine learning models, particularly in neural network models. Although IG is always utilized for image classification to visualize important input features, we selected IG to interpret a time-series binary classification model to visualize important input features because IG can apply to a machine learning model without modifying the original deep neural network.

The methods of IG implementation in the time-series binary classification model are the same as image classification. First, baseline time-series input (zero-initialized time-series) is divided in equally spaced intermediate steps by actual time-series input. For each intermediate step, compute the gradient of the model’s output with respect to the input. After that, the computed gradients over all the intermediate steps are integrated. The integrated gradients for each input feature indicate the contribution of that feature to the final prediction. Therefore, the important input features in the time-series result are expressed. However, time series cannot be visualized for easy understanding as images with IG mapping. We decided to calculate the mean of IG feature values to visualize the impacts of features and calculate the absolute mean for arranging the impacts in order.

## 4. Results and Discussion

Table 4 displays the results of all fusion strategies for experiment modals. As a consequence, the Window Block LSTM model experiment using the intermediate fusion strategy gave the best results overall, with an accuracy of 0.8958.

Since there is human error in depression analysis, particularly for mild and moderate depression levels that can be distinguished between depressed and non-depressed individuals, Window Block LSTM and Bi-LSTM models that employ intermediate fusion techniques were tested with label smoothing. The results of Table 5 demonstrate that both models perform better when label smoothing is configured properly. With (0.3, 0.7) label smoothing, the accuracy of the Bi-LSTM model is 0.9167 with a zero false positive value. The highest F1-score is achieved by the Window Block LSTM with (0.05, 0.95) label smoothing, which also has an accuracy of 0.9167. We intend to ensure that our Window Block LSTM performs well on other data sets. Therefore, we tested our model with additional data, which is shown in Appendix A.

We experimented with Bi-LSTM [18] and transformer models [19] in our data set to evaluate our model using solely facial features. Compared to baseline, experimental versions of the Bi-LSTM and Window Block LSTM models perform better, as shown in Table 6. Our Window Block LSTM model surpasses the baseline with an accuracy of 91.67% and a macro F1-score of 88.89%. similar to our Bi-LSTM, which has an accuracy rate of 91.67% and a macro F1-score rate of 88.21%.

**Figure 9:**
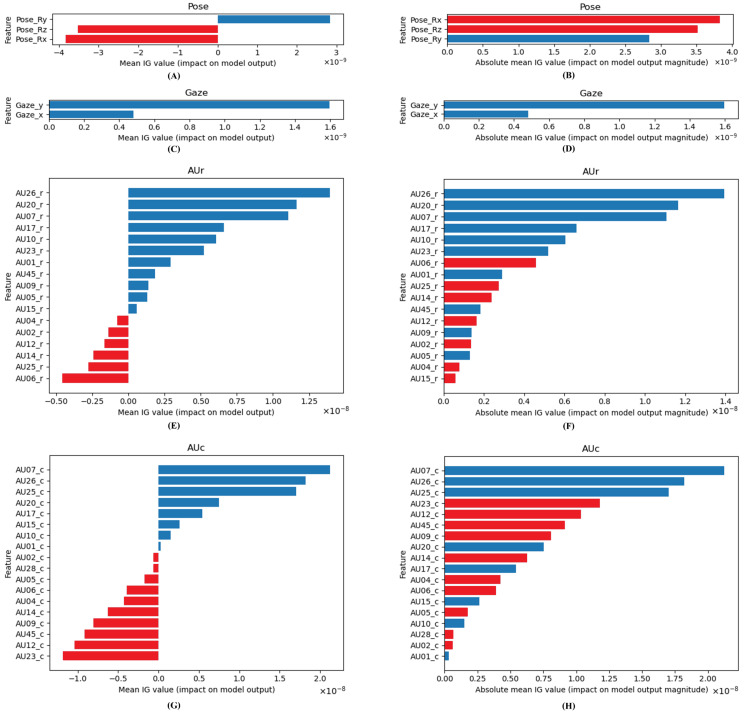
(**A**) Pose impact on model output, (**B**) Pose impact on model output magnitude, (**C**) Gaze impact on model output, (**D**) Gaze impact on model output magnitude, (**E**) AUr impact on model output, (**F**) AUr impact on model output magnitude, (**G**) AUc impact on model output, (**H**) AUc impact on model output magnitude. *Red color refers to a negative effect (tends to be non-depressive), and blue color refers to a positive effect (tends to be depressive)*.

**Figure 10:**
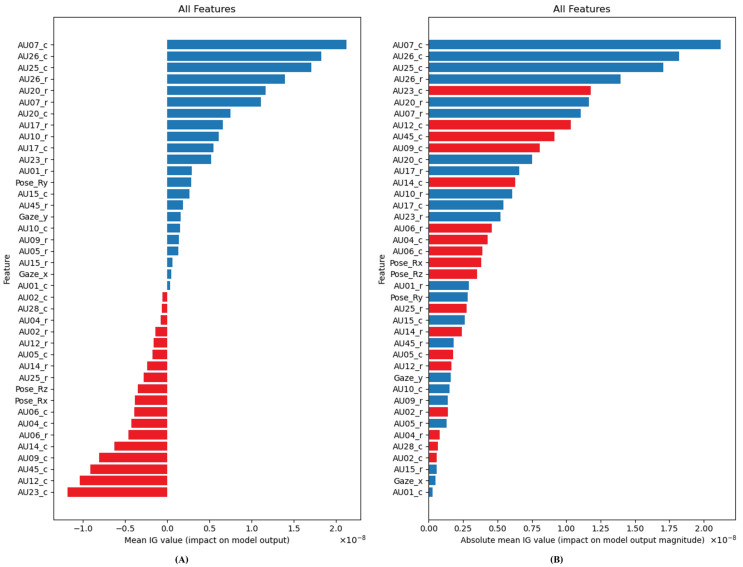
(**A**) Positive/negative impact of all features. (**B**) Absolute impact (magnitude) of all features. *Red color refers to a negative effect (tends to be non-depressive), and blue color refers to a positive effect (tends to be depressive)*.

Important key features that represent depressive symptoms are shown in Figure 9. The four main features are separated for apparent visualization. Each feature contains two graphs that represent the important key features of depression or non-depression and their order of importance. (A), (C), (E), and (G) display the impact on output of pose, gaze, action unit regression, and action unit classification, respectively. (B), (D), (F), and (H) display the importance of pose, gaze, action unit regression, and action unit classification, respectively. Finally, a summary of all features is shown in Figure 10 by containing two graphs that represent the important key features and their order of importance.

**Figure 11:**
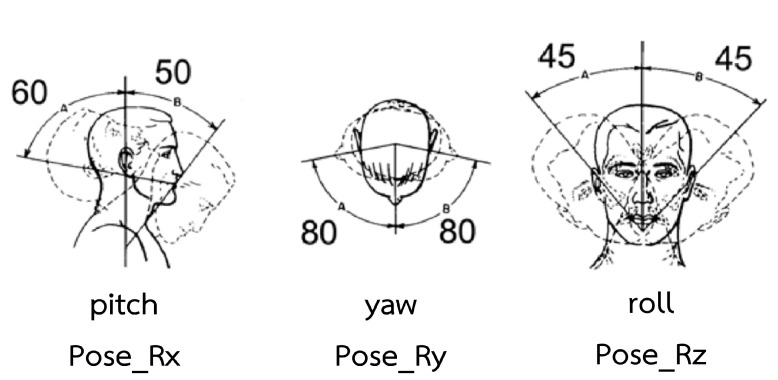
Head pose movement [30].

Crucial pose features are pitch, roll, and yaw, respectively, as shown in Figure 9B. The movement of head pose features is shown in Figure 11. Figure 9A shows that pitch and roll influence model to predict non-depression because head-nodding and head tilting indicate high energy and favorable to social interaction [26,27,28,29]. On the other hand, yaw has an impact on depression because head turning means a lack of concentration on social interests and withdrawal.

**Figure 12:**
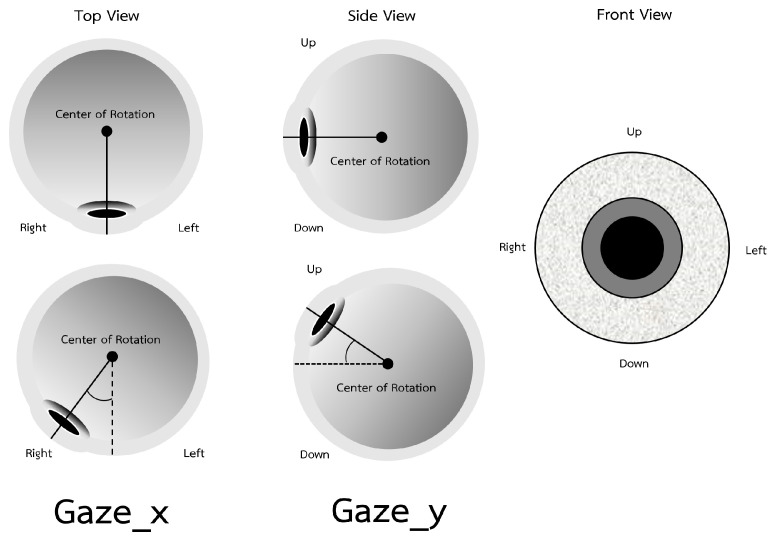
Gaze movement.

Significant gaze features are looking up or down and looking left or right, as shown in Figure 9D. The movement of eye gaze features is shown in Figure 12. Figure 9C shows that both gaze features have an impact on depression because looking around means patients are not concentrating and are absent-minded. Following previous studies in [15], the reduction in eye movement can be observed by a model to determine depressive symptoms. Moreover, nonspecific gaze and not having eye contact can be classified as gestures of depression [26,27,28].

Figure 9E,F show the impact on depression or non-depression of action unit regression features and the important order of action unit regression features, respectively. The movement of the action unit is shown in Figure 1. The obvious features that relate to depression are the AU26 jaw drop, AU20 lip stretcher, and AU07 lid tightener, which represent grumbling, frowning, and scowling faces. All three outstanding features express negative feelings and social disinterest [28]. On the other hand, the features that relate to non-depression are the AU06 cheek raiser, AU25 lips part, AU14 dimpler, and AU12 lip corner puller, which represent the posture of talking and smiling. This means people have social interest and happiness as non-depression.

The impact on depression or non-depression of action unit classification and the important order of action unit classification are shown in Figure 9G,H, respectively. The movement of the action unit is shown in Figure 1. In the same direction of action unit regression, AU07 lid tightener and AU26 jaw drop are related to depression. Conversely, the AU25 lips part of action unit regression has an impact on non-depression. In the action unit classification, the AU25 lips part has an impact on depression because this action unit represents when people talk. In the same direction as [28], silence and speaking can be classified as depression or non-depression depending on speech content. The features that relate to non-depression are the AU23 lip tightener, the AU12 lip corner puller, the AU45 blink, and the AU09 nose wrinkler, which represent the expression of a social interest—pursing lips, smiling, and blinking, for instance.

Finally, the summary of all features is shown in Figure 10. The important features are action unit classification, action unit regression, head pose, and gaze, respectively. Both action unit classification and regression are the indicators to classify depression because facial expression can be detected from them as human observation [14]. Head pose and gaze are indicators that can detect concentration and social interest. Therefore, depression can be detected by four main features that are explainable.

The explainable model is helpful for understanding how the model works. The insight that we found is that depressive patients have a lack of concentration, social disinterest, and negative feelings. They are inclined to turn their heads, gaze in no specific direction, and move their eyes slower than normal people. They do not smile and always keep grumbling, frowning, and scowling.

## 5. Conclusions

Our paper aimed to improve model accuracy by experimenting with three types of fusion model techniques that use the transformer model, window block LSTM, and Bi-LSTM with feature selection techniques and label smoothing techniques. The best model for depression detection is the intermediate window block LSTM model with appropriate label smoothing (0.9, 0.1). The model obtains a 88.89% F1-score, 87.03% recall, 91.67% accuracy, and 91.40% precision. In addition, the model achieves 100% accuracy for the mild and severe classes. The advantages of this model in comparison to other experiment models are that window blocks can train faster than Bi-LSTM models and can continue to pass through lengthy time series data. The model can represent the significant facial features that are easily understood after being run using the integrated gradient technique on a test data set. This may prompt the creation of a tool that concentrates on important parts of videos in order to speed up the diagnosis process for psychiatrists.

## Figures and Tables

**Table 1:** Related works of depression prediction. * CCC refers to concordance correlation coefficient.

Year	Techniques	Data Sets	Questionnaires	Accuracy	Precision	Recall	F1 Score
2019 [18]	Multi-Model, Bi-LSTM	E-DAIC	PHQ-8	CCC * 0.670	-	-	-
2021 [19]	Multi-Modal Transformer	E-DAIC	PHQ-8	CCC * 0.733	-	-	-
2021 [20]	PSO, FFNN	DAIC WOZ	PHQ-8	0.978	-	-	-
2022 [21]	FDR, ILDA	DAIC WOZ	PHQ-8	-	-	-	0.805
2022 [22]	FFNN	Their own	-	0.860	0.830	0.820	0.820

**Table 2:** Data Distribution.

Data Set	Non-Depression	Depression	Normal	Mild	Moderate	Severe
Train Data Set	270	108 × 2 = 216	84	186	90 × 2 = 180	18 × 2 = 36
Validation Data Set	35	13	11	24	11	2
Test Data Set	35	13	11	24	11	2

**Table 3:** Preliminary experiment with each feature. *Highlighted numbers refer to the winners*.

Model		TP	FN	TN	FP	Acc (%)	Macro (%)			Micro (%)		
							**Precision**	**Recall**	**F1**	**Precision**	**Recall**	**F1**
Bi-LSTM	PoseR	6	7	32	3	79.17	74.36	68.79	70.52	77.88	79.17	77.84
	PoseL	5	8	33	2	79.17	75.96	66.37	68.42	78.03	79.17	76.86
	GazeR	8	5	31	4	81.25	76.39	75.05	75.66	80.84	81.25	81.01
	GazeV	7	6	32	3	81.25	77.11	72.64	74.27	80.36	81.25	80.41
	AUr	7	6	33	2	83.33	81.20	74.07	76.41	82.76	83.33	82.27
	AUc	8	5	33	2	85.42	83.42	77.91	79.99	84.99	85.42	84.77

**Table 4:** Fusion modality result comparison. *Highlighted numbers refer to the winners*.

Model		TP	FN	TN	FP	Acc (%)	Macro (%)			Micro (%)		
							**Precision**	**Recall**	**F1**	**Precision**	**Recall**	**F1**
Bi-LSTM	Early Fusion	6	7	32	3	79.17	74.36	68.79	70.52	77.88	79.17	77.84
	Intermediate Fusion	6	7	35	0	85.42	91.67	73.08	77.03	87.85	85.42	83.39
	Late Fusion	7	6	33	2	83.33	81.20	74.07	76.41	82.76	83.33	82.27
Window Block LSTM	Early Fusion	8	5	31	4	81.25	76.39	75.05	75.66	80.84	81.25	81.01
	Intermediate Fusion	10	3	33	2	89.58	87.50	85.60	86.48	89.41	89.58	89.45
	Late Fusion	7	6	33	2	83.33	81.20	74.07	76.41	82.76	83.33	82.27
Transformer	Early Fusion	5	8	32	3	77.08	71.25	64.95	66.48	75.26	77.08	75.12
	Intermediate Fusion	7	6	33	2	83.33	81.20	74.07	76.41	82.76	83.33	82.27
	Late Fusion	7	6	32	3	81.25	77.11	72.64	74.27	80.36	81.25	80.41

**Table 5:** Intermediate fusion with label smoothing result comparison. *Highlighted numbers refer to the winners*.

**Model**		**TP**	**FN**	**TN**	**FP**	**Acc (%)**	Macro (%)			Micro (%)		
							**Precision**	**Recall**	**F1**	**Precision**	**Recall**	**F1**
Bi-LSTM	(0, 1)	6	7	35	0	85.42	91.67	73.08	77.03	87.85	85.42	83.39
	(0.05, 0.95)	5	8	33	2	79.17	75.96	66.37	68.42	78.03	79.17	76.86
	(0.1, 0.9)	6	7	33	2	81.25	78.75	70.22	72.57	80.47	81.25	79.64
	(0.15, 0.85)	7	6	33	2	83.33	81.20	74.07	76.41	82.76	83.33	82.27
	(0.2, 0.8)	6	7	32	3	79.17	74.36	68.79	70.52	77.88	79.17	77.84
	(0.25, 0.75)	5	8	34	1	81.25	82.14	67.80	70.47	81.60	81.25	78.65
	(0.3, 0.7)	9	4	35	0	91.67	94.87	84.62	88.21	92.52	91.67	91.13
	(0.35, 0.65)	5	8	34	1	81.25	82.14	67.80	70.47	81.60	81.25	78.65
	(0.4, 0.6)	5	8	34	1	81.25	82.14	67.80	70.47	81.60	81.25	78.65
	(0.45, 0.55)	6	7	34	1	83.33	84.32	71.65	74.74	83.68	83.33	81.49
Window Block LSTM	(0, 1)	10	3	33	2	89.58	87.50	85.60	86.48	89.41	89.58	89.45
	(0.05, 0.95)	10	3	34	1	91.67	91.40	87.03	88.89	91.63	91.67	91.44
	(0.1, 0.9)	7	6	32	3	81.25	77.11	72.64	74.27	80.36	81.25	80.41
	(0.15, 0.85)	6	7	33	2	81.25	78.75	70.22	72.57	80.47	81.25	79.64
	(0.2, 0.8)	4	9	34	1	79.17	79.53	63.96	65.81	79.32	79.17	75.61
	(0.25, 0.75)	6	7	32	3	79.17	74.36	68.79	70.52	77.88	79.17	77.84
	(0.3, 0.7)	6	7	34	1	83.33	84.32	71.65	74.74	83.68	83.33	81.49
	(0.35, 0.65)	6	7	33	2	81.25	78.75	70.22	72.57	80.47	81.25	79.64
	(0.4, 0.6)	7	6	32	3	81.25	77.11	72.64	74.27	80.36	81.25	80.41
	(0.45, 0.55)	6	7	34	1	85.42	86.25	75.49	78.67	85.68	85.42	84.17

**Table 6:** Baseline and our result comparison *Highlighted numbers refer to the winners*.

**Model**	**TP**	**FN**	**TN**	**FP**	**Acc (%)**	Macro (%)			Micro (%)		
						**Precision**	**Recall**	**F1**	**Precision**	**Recall**	**F1**
Bi-LSTM Baseline	0	13	33	2	68.75	35.87	47.14	40.74	52.31	68.75	59.41
Transformer Baseline	5	8	28	7	68.75	59.72	59.23	59.44	68.00	68.75	68.35
Our Bi-LSTM	9	4	35	0	91.67	94.87	84.62	88.21	92.52	91.67	91.13
Our Window Block LSTM	10	3	34	1	91.67	91.40	87.03	88.89	91.63	91.67	91.44

## Data Availability

The data are not publicly available due to privacy restrictions.

## Appendix A. Results from an Additional Data Set

The additional data set was collected in different periods of the main study with the same protocol. The data set contains 20 normal, 10 mild, 10 moderate, and 10 severe depressive levels. The results of our window block LSTM are shown in Table A1. Our window block LSTM with label smoothing has the best performance with an 77.72% macro F1-score. In the same direction as the main study, our window block LSTM has the best result, and the secondary is our Bi-LSTM. Both our models have better performance than the Bi-LSTM baseline and the transformer baseline.

**Table A1:** Additional data set results comparison. *Highlighted numbers refer to the winners*.

**Model**	**TP**	**FN**	**TN**	**FP**	**Acc (%)**	Macro (%)			Micro (%)		
						**Precision**	**Recall**	**F1**	**Precision**	**Recall**	**F1**
Bi-LSTM Baseline	0	20	30	0	60.00	30.00	50.00	37.50	36.00	60.00	45.00
Transformer Baseline	0	20	30	0	60.00	30.00	50.00	37.50	36.00	60.00	45.00
Our Bi-LSTM	9	11	28	2	74.00	76.81	69.17	69.61	75.80	74.00	71.92
Our Window Block LSTM	12	8	28	2	80.00	81.75	76.67	77.72	80.95	80.00	79.14

## References

[1] Santomauro D.F., Herrera A.M.M., Shadid J., Zheng P., Ashbaugh C., Pigott D.M., Abbafati C., Adolph C., Amlag J.O., Aravkin A.Y. (2021). Global prevalence and burden of depressive and anxiety disorders in 204 countries and territories in 2020 due to the COVID-19 pandemic. Lancet.

[2] Hawes M.T., Szenczy A.K., Klein D.N., Hajcak G., Nelson B.D. (2022). Increases in depression and anxiety symptoms in adolescents and young adults during the COVID-19 pandemic. Psychol. Med..

[3] Tabur A., Elkefi S., Emhan A., Mengenci C., Bez Y., Asan O. (2022). Anxiety, burnout and depression, psychological well-being as predictor of healthcare professionals’ turnover during the COVID-19 pandemic: Study in a pandemic hospital. Healthcare.

[4] Calegaro V.C., Ramos-Lima L.F., Hoffmann M.S., Zoratto G., Kerber N., Costa F.C.D., Picinin V.D., Köchler J., Rodrigues L., Maciel L. (2022). Closed doors: Predictors of stress, anxiety, depression, and PTSD during the onset of COVID-19 pandemic in Brazil. J. Affect. Disord..

[5] Cheng X., Wang Q., Wang R., Wang Y., Chen X., Mi G., Chen X., Wang L., Wang C., Hu L. (2022). Prevalence of depressive disorders and associated demographic characteristics in Shandong: An epidemiological investigation. J. Affect. Disord..

[6] Smith K.M., Renshaw P.F., Bilello J. (2013). The diagnosis of depression: Current and emerging methods. Compr. Psychiatry.

[7] Ekman P., Friesen W.V. (1978). Facial action coding system. Environmental Psychology & Nonverbal Behavior.

[8] Baltrušaitis T., Robinson P., Morency L.P. Openface: An open source facial behavior analysis toolkit. Proceedings of the 2016 IEEE winter conference on applications of computer vision (WACV).

[9] Sundararajan M., Taly A., Yan Q. Axiomatic attribution for deep networks. Proceedings of the International Conference on Machine Learning, PMLR.

[10] Watanabe A., Yamazaki T. (2021). Representation of the brain network by electroencephalograms during facial expressions. J. Neurosci. Methods.

[11] Fu C.H., Williams S.C., Brammer M.J., Suckling J., Kim J., Cleare A.J., Walsh N.D., Mitterschiffthaler M.T., Andrew C.M., Pich E.M. (2007). Neural responses to happy facial expressions in major depression following antidepressant treatment. Am. J. Psychiatry.

[12] Nakamura A., Yomogida Y., Ota M., Matsuo J., Ishida I., Hidese S., Kunugi H. (2022). The cerebellum as a moderator of negative bias of facial expression processing in depressive patients. J. Affect. Disord. Rep..

[13] Schirmer A., Adolphs R. (2017). Emotion perception from face, voice, and touch: Comparisons and convergence. Trends Cogn. Sci..

[14] Scott N.J., Kramer R.S.S., Jones A.L., Ward R. (2013). Facial cues to depressive symptoms and their associated personality attributions. Psychiatry Res..

[15] Stolicyn A., Steele J.D., Seriès P. (2022). Prediction of depression symptoms in individual subjects with face and eye movement tracking. Psychol. Med..

[16] Liu G.D., Li Y.C., Zhang W., Zhang L. (2020). A brief review of artificial intelligence applications and algorithms for psychiatric disorders. Engineering.

[17] Ringeval F., Schuller B., Valstar M., Cummins N., Cowie R., Tavabi L., Schmitt M., Alisamir S., Amiriparian S., Messner E.M. AVEC 2019 workshop and challenge: State-of-mind, detecting depression with AI, and cross-cultural affect recognition. Proceedings of the 9th International on Audio/Visual Emotion Challenge and Workshop.

[18] Ray A., Kumar S., Reddy R., Mukherjee P., Garg R. Multi-level attention network using text, audio and video for depression prediction. Proceedings of the 9th International on Audio/Visual Emotion Challenge and Workshop.

[19] Sun H., Liu J., Chai S., Qiu Z., Lin L., Huang X., Chen Y. (2021). Multi-modal adaptive fusion transformer network for the estimation of depression level. Sensors.

[20] Akbar H., Dewi S., Rozali Y.A., Lunanta L.P., Anwar N., Anwar D. Exploiting facial action unit in video for recognizing depression using metaheuristic and neural networks. Proceedings of the 2021 1st International Conference on Computer Science and Artificial Intelligence (ICCSAI).

[21] Rathi S., Kaur B., Agrawal R. (2022). Selection of relevant visual feature sets for enhanced depression detection using incremental linear discriminant analysis. Multimed. Tools Appl..

[22] Schultebraucks K., Yadav V., Shalev A.Y., Bonanno G.A., Galatzer-Levy I.R. (2022). Deep learning-based classification of posttraumatic stress disorder and depression following trauma utilizing visual and auditory markers of arousal and mood. Psychol. Med..

[23] Serengil S.I., Ozpinar A. HyperExtended LightFace: A Facial Attribute Analysis Framework. Proceedings of the 2021 International Conference on Engineering and Emerging Technologies (ICEET).

[24] Hamilton M. (1960). A rating scale for depression. J. Neurol. Neurosurg. Psychiatry.

[25] Müller R., Kornblith S., Hinton G.E. (2019). When does label smoothing help?. Adv. Neural Inf. Process. Syst..

[26] Fossi L., Faravelli C., Paoli M. (1984). The ethological approach to the assessment of depressive disorders. J. Nerv. Ment. Dis..

[27] Schelde J.T.M. (1998). Major depression: Behavioral markers of depression and recovery. J. Nerv. Ment. Dis..

[28] Fiquer J.T., Boggio P.S., Gorenstein C. (2013). Talking bodies: Nonverbal behavior in the assessment of depression severity. J. Affect. Disord..

[29] Gahalawat M., Fernandez Rojas R., Guha T., Subramanian R., Goecke R. Explainable Depression Detection via Head Motion Patterns. Proceedings of the International Conference on Multimodal Interaction.

[30] Doss A.S.A., Lingampally P.K., Nguyen G.M.T., Schilberg D. (2023). A comprehensive review of wearable assistive robotic devices used for head and neck rehabilitation. Results Eng..

